# Increased neutrophil-to-lymphocyte ratio is associated with disease-specific mortality in patients with penile cancer

**DOI:** 10.1186/s12885-016-2443-6

**Published:** 2016-07-07

**Authors:** Jun Kasuga, Takashi Kawahara, Daiji Takamoto, Sachi Fukui, Takashi Tokita, Tomoyuki Tadenuma, Masaki Narahara, Syusei Fusayasu, Hideyuki Terao, Koji Izumi, Hiroki Ito, Yusuke Hattori, Jun-ichi Teranishi, Takeshi Sasaki, Kazuhide Makiyama, Yasuhide Miyoshi, Masahiro Yao, Yasushi Yumura, Hiroshi Miyamoto, Hiroji Uemura

**Affiliations:** Department of Urology, Yokohama City University Graduate School of Medicine, 3-9 Fukuura, Kanazawa-ku, Yokohama, Kanagawa 2360004 Japan; Departments of Urology and Renal transplantation, Yokohama City University Medical Center, Yokohama, Japan; Department of Urology, Yokohama Minami Kyosai Hospital, Yokohama, Japan; Department of Urology, Yokohama Minato Red Cross Hospital, Yokohama, Japan; Department of Urology, Yokosuka Kyosai Hospital, Yokosuka, Japan; Department of Urology, Kanagawa Cancer Center, Yokohama, Japan; Department of Urology, International Goodwill Hospital, Yokohama, Japan; Department of Urology, Yamato Municipal Hospital, Yamato, Japan; Department of Urology, Fujisawa City Hospital, Fujisawa, Japan; Department of Urology, Yokohama Municipal Citizen’s Hospital, Yokohama, Japan; Department of Pathology, The University of Tokyo Graduate School of Medicine, Tokyo, Japan; Departments of Pathology and Urology, Johns Hopkins University School of Medicine, Baltimore, USA

**Keywords:** Penile cancer, Biomarker, Neutrophil-to-lymphocyte ratio, Immunohistochemistry

## Abstract

**Background:**

The neutrophil-to-lymphocyte ratio (NLR), a simple marker of the systemic inflammatory response, has been demonstrated to correlate with patient outcomes for various solid malignancies. We investigated the utility of the pretreatment NLR as a prognosticator in patients who presented with penile cancer.

**Methods:**

A total of 41 patients who underwent complete blood count with differential and subsequent radical penectomy from 1988 to 2014 were analyzed. We assessed the correlation between the NLR and the prognosis of penile cancer.

**Results:**

The median and mean (± SD) NLRs in 41 penile cancer patients were 3.42 and 5.03 ± 4.99, respectively. Based on the area under receiver operator characteristic curve, the cut-off value of NLR was determined to be 2.82. Patients with a high NLR (≥2.82) showed a significantly poorer cancer-specific survival (*p* = 0.023) than those with a low NLR.

**Conclusions:**

The pretreatment NLR may function as a biomarker that precisely predicts the prognosis in patients with penile cancer.

## Background

Penile squamous cell carcinoma (PSCC) is a rare disease in developed countries, with an incidence of 0.3–1.0 per 100,000 males in Europe and North America and 0.4–0.5 per 100,000 males in Japan [1, 2]. However, it represents an important public health problem for developing countries in Asia, Africa and South America, where its incidence varies from 3 to 8.3 cases per 100,000 [3]. The major prognostic factors in PSCC are tumor grade and the presence of perineal and lymphatic invasion [4]. SCC, a soluble epithelial antigen and a classical molecular marker lacks sensitivity in the detection of small tumor burdens and has little prognostic significance in survival after surgery [5]. The overexpression of p53 and Ki-67 and the loss of membranous E-cadherin determined immunohistochemically in biopsy or penectomy tissue specimens are also shown to associate with the detection of lymph node metastases, but these markers are not useful in clinical practice [4, 6].

The neutrophil-to-lymphocyte ratio (NLR) has been suggested as a simple marker of the systemic inflammatory response in critical care patients [7]. It has also been reported as an independent prognostic factor for several solid malignancies [8–17]. Importantly, the NLR can easily be calculated from routine complete blood counts (CBCs) in peripheral blood samples [15, 16].

We investigated the utility of the pretreatment NLR as a prognosticator in patients who presented with penile cancer.

## Methods

### Patients

**Fig. 1:**
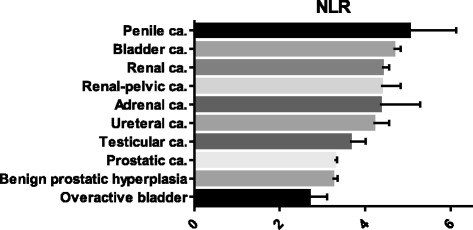
The NLRs in patients with urological diseases. Each value represents the mean and 95 % confidence interval

A total of 73,637 CBC exams, which included absolute neutrophil and lymphocyte counts, were performed in 9782 male patients at the Department of Urology, Yokohama City University Hospital (Yokohama, Japan) from 1999 to 2015. Among these, we investigated the NLRs in patients with urological diseases, such as penile cancer, renal cell carcinoma, prostate cancer, testicular cancer, overactive bladder, and benign prostatic hyperplasia (Fig. 1).

We conducted a retrospective study of 41 patients with penile cancer who underwent CBCs and subsequent radical penectomy at Yokohama City University Medical Center and 24 other institutions from 1988 to 2014. Some of the patients who were diagnosed at Yokohama City University Hospital overlapped with those in a study of 9782 male patients. All of the patients were pathologically diagnosed with PSCC. Patients were followed-up for 2.3–271.7 months (median: 34.7 months). The incidence of tumor relapse and patient outcomes during the follow-up period were evaluated. This study was approved by the ethics committee of Yokohama City University, Yokohama City University Medical Center and the other participating institutions approved the protocol of the present study. Written informed consent was obtained from the penile cancer patients.

### Clinical and laboratory assessments

The NLR was calculated using the neutrophil and lymphocyte counts, which were obtained via CBCs before the surgery. We determined the cut-off point of the NLR according to the sensitivity and specificity levels derived from area under receiver operator characteristic (AUROC) curve.

### Immunohistochemistry

An immunohistochemical analysis was performed in 5-μm-thick tissue microarray (TMA) sections, including 183 penile tissue specimens that were obtained from US Biomax (PE2081, Rockville, MD). Immunohistochemical staining was performed., as described previously [18], using a primary antibody to CD66b (clone G10F5, diluted to 1:200, BD Biosciences, San Jose, CA, USA) or CD8 (clone C8/144B, diluted to 1:100, DAKO Corporation, Carpenteria, CA, USA), to detect tumor-infiltrating neutrophils and lymphocytes, respectively. The slides were then examined by a single pathologist (HM) who was blinded to the identity of the samples. The total numbers of CD66b-positive and CD8-positive cells were counted in each TMA core. Due to the small number benign specimens and some of the specimens including infection, we did not compare between benign and malignant specimens.

### Statistical analyses

The patients’ characteristics and preoperative factors were analyzed using the Mann–Whitney *U* and chi-squared tests, using the Graph Pad Prism software program (Graph Pad Software, La Jolla, CA, USA). The survival duration was defined as the time between the dates of pathological diagnosis and tumor progression or death. A log-rank test was performed for comparison between higher and lower NLR groups. Multivariate logistic regression models were used to detect the individual factors. *P* values of <0.05 were considered to indicate statistical significance.

## Results

### Penile cancer patients showed a higher NLR

According to the clinical database of the Department of Urology at Yokohama City University Hospital, 9782 patients underwent an NLR check during the study period. The median/mean NLRs for each disease (mainly the names of the diagnosed diseases which were used for medical insurance) were 3.42/5.03 (penile cancer), 2.67/4.67 (bladder cancer), 2.64/4.40 (renal cell carcinoma), (2.39/4.37 (renal pelvic cancer), 3.58/4.35 (adrenal cancer), 2.51/4.20 (ureteral cancer), 2.39/3.65 (testicular cancer), 2.26/3.28 (prostatic cancer), 2.22/3.23 (benign prostatic hyperplasia), and 2.41/2.68 (overactive bladder) (Fig. 1). The NLR of the PSCC patients was significantly higher than the NLRs of testicular cancer (*p* <0.05), prostate cancer (*p* <0.01), benign prostate hyperplasia (*p* < 0.01), and overactive bladder (*p* <0.001) patients.

### The NLR predicts the survival of penile cancer patients

The median and mean (± SD) ages of the 41 patients were 69 and 68.5 (±11.4) years with median and mean (± SD) follow-up periods of 34.7 and 60.7 (±52.1) months after the initial diagnosis. The clinicopathological data of these patients are summarized in Table 1.

**Table 1:** Patients’ characteristics

Variables	number or median (mean ± SD)			*p* value
	all	NLR < 2.82	NLR ≥ 2.82	
A number of patients.	41 (100.0 %)	21 (51.2 %)	20 (48.8 %)	
Age (years)	69 (68.5 ± 11.8)	69 (66.3 ± 10.9)	71 (70.7 ± 12.5)	0.243
Location				
Gland	30 (73.2 %)	15 (57.1 %)	15 (75.0 %)	0.287
Foreskin	8 (19.5 %)	5 (23.8 %)	3 (15.0 %)	
Shaft	2 (4.9 %)	0 (0.0 %)	2 (10.0 %)	
Unknown	1 (2.4 %)	1 (4.8 %)	0 (0.0 %)	
Tumor grade (differentiation)				
Well	26 (63.4 %)	15 (57.1 %)	11 (55.0 %)	0.330
Moderate	10 (24.0 %)	3 (14.3 %)	7 (35.0 %)	
Poor	2 (4.9 %)	1 (4.8 %)	1 (5.0 %)	
Unknown	3 (7.3 %)	2 (9.5 %)	1 (5.0 %)	
Pathological T stage				
1	22 (53.7 %)	14 (66.7 %)	8 (40.0 %)	0.273
2	13 (31.’%)	4 (19.0 %)	9 (45.0 %)	
3	2 (4.9 %)	1 (4.8 %)	1 (5.0 %)	
4	3 (7.3 %)	1 (4.8 %)	2 (10.0 %)	
Unknown	1 (2.4 %)	1 (4.8 %)	0 (0.0 %)	
Lymph node metastasis	9 (21.6 %)	2 (9.5 %)	7 (35.0 %)	0.049
Distant metastasis	1 (2.4 %)	1 (4.8 %)	0 (0.0 %)	0.323
Anatomic stage				
I	17 (41.5 %)	10 (47.6 %)	7 (35.0 %)	0.214
II	14 (34.1 %)	8 (38.1 %)	6 (30.0 %)	
III	3 (7.3 %)	0 (0.0 %)	3 (15.0 %)	
IV	6 (14.6 %)	2 (9.5 %)	4 (20.0 %)	
Unknown	1 (2.4 %)	1 (4.8 %)	0 (0.0 %)

**Table 2:** Tumor-infiltrating CD66b/CD8-positive immune cells in the penile TMA (PE2081)

	Pathological Grade			*p* value
	Grade 1 (*n* = 128)	Grade 2&3 (*n* = 47)	Unknown (*n* = 8)	
CD66b	15 (23.8 ± 25.5)	12 (31.8 ± 38.0)	7.5 (14.4 ± 14.1)	0.209
CD8	62 (58.3 ± 37.1)	60.5 (56.8 ± 37.5)	68.5 (59.9 ± 32.0)	0.898
Anatomic Stage				
	Stage I (*n* = 147)	Stage II & III (*n* = 36)		
CD66b	16 (28.7 ± 31.2)	7 (15.9 ± 19.9)		0.014
CD8	65 (60.0 ± 36.5)	35 (49.1 ± 38.2)		0.183

Data represent the median (mean ± SD)

**Fig. 2:**
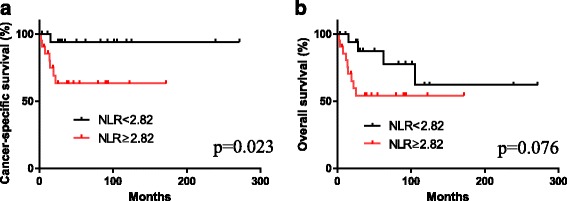
The correlation between the NLR and **a** cancer-specific survival or **b** overall survival

We created an AUROC curve to determine the NLR cut-off value for predicting the prognosis of penile cancer patients. The cut-off value was determined to be 2.82. High NLR was significantly associated with lymph node metastasis (*p* = 0.049). Patients with high NLRs also showed a significantly poorer cancer-specific survival (*p* = 0.023) than those with low NLRs (Fig. 2a). In addition, patients with high NLRs tended to correlate with poorer overall survival (*p* = 0.076) (Fig. 2b). We performed a multivariate analysis but it did not reveal any significant independent factors that predicted the prognosis.

### Tumor-infiltrating neutrophils and lymphocytes in PSCC

**Fig. 3:**
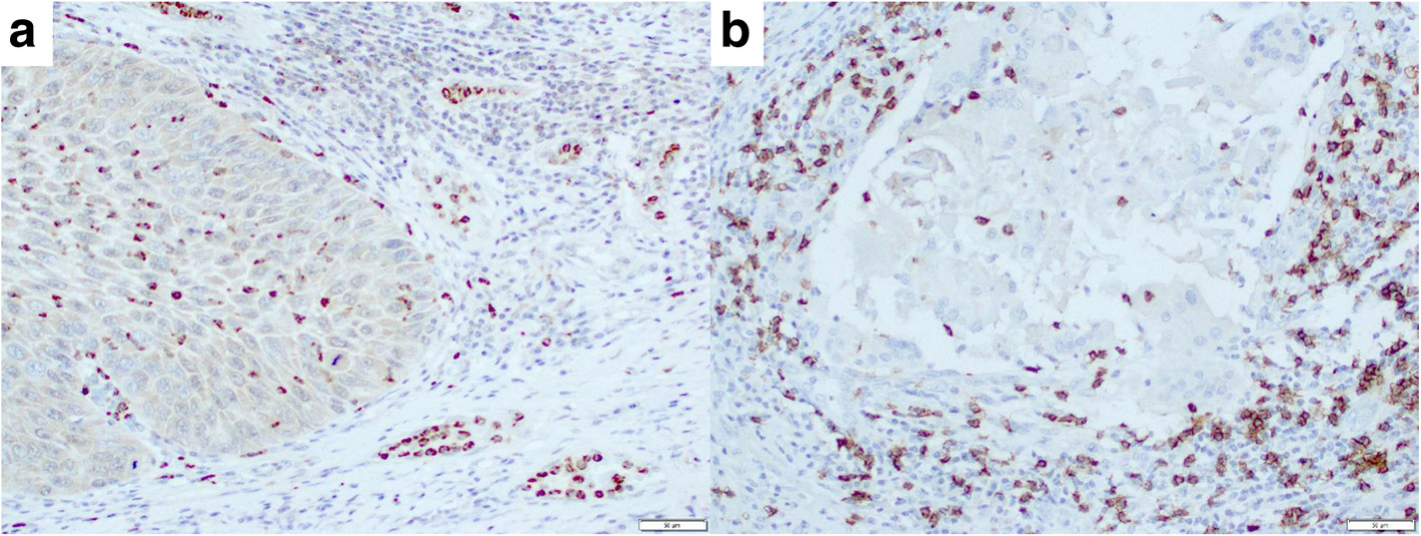
Immunohistochemistry of **a** CD66b and **b** CD8 in penile cancer specimens. CD66b- and CD8-positive immune cells are seen predominantly in the stromal tissue

Immunohistochemistry was used to determine the numbers of tumor-infiltrating neutrophils and lymphocytes in the penile TMA. Both CD66b- and CD8-positive immune cells were present in the specimens (Fig. 3). We then analyzed the relationship between the number of tumor-infiltrating CD66b-positive neutrophils or CD8-positive lymphocytes and tumor grade or stage. There were no statistically significant correlations between the number of CD66b- or CD8-positive cells and tumor grade. Interestingly, the number of CD66b-positive cells, but not that of CD8-positive cells, was significantly lower in high stage disease, compared with stage 1 disease (*p* = 0.014). However, there were no statistically significant differences in the ratio of CD66b/CD8-positive cell Table 2.

## Discussion

We have evaluated the pretreatment NLR as a predictor of survival in penile cancer patients. We found that high NLRs were associated with a poorer prognosis of penile cancer.

Several prognostic factors have been established for patients with penile cancer. Nodal metastasis is the most important predictor of a poor clinical outcome [4]. Tumor grade and perineural or lymphatic invasion are also known prognostic predictors. p53, Ki-67, E-cadherin, and epidermal growth factor receptor (EGFR) are considered to be molecular prognostic markers, but they are not always useful in clinical practice [4, 6].

It has been suggested that the NLR can be used to estimate the magnitude of systemic inflammation in cancer patients [8, 18–20]. The NLR is easily and inexpensively measured [21]. An elevated NLR has been reported to be associated with a poorer survival rate in a variety of cancers [8–13, 22].

Pond et al. demonstrated that the NLR was significantly associated with survival in 26 patients with penile cancer [21]. This has been the only paper to describe the relationship between the NLR and the prognosis of penile cancer; however, the subjects were limited to patients who were undergoing concurrent chemo-radiotherapy.

The AUROC determined the cut-off value of the NLR to be 2.82 in the present study. Several studies in patients with advanced pancreatic cancer have shown NLR cut-off values of approximately five [8]. In patients with intrahepatic cholangiocarcinoma and those with liver metastasis from colorectal carcinoma [9], the NLR cut-off value is also set at five. In urological cancers, an NLR cut-off value of approximately five has been used for prostate cancer, while scores of 2 to 5 have been used for renal cell carcinoma [23]. Our cut-off point for the NLR was thus somewhat lower than the values determined in previous studies, despite the fact that the NLR for penile cancer was high in comparison to other urological diseases. Of note, in most of these studies [ref], the NLR was assessed in advanced cases. On the other hand, in our prevous study [24], the NLR cut-off point for predicting the prognosis of patients most of who had organ-confined prostate cancer was 2.4.

In recent studies, the preoperative levels of C-reactive protein (CRP) were found to predict survival in patients with penile squamous cell carcinoma [24, 25]. In various tumors, other markers of the systemic inflammatory response have also been developed to predict patient outcomes, such as the platelet-to-lympocyte ratio (PLR), the lympocyte-to-monocyte ratio (LMR), and the preoperative haemogolobin and albumin levels [26–31]. It is necessary to investigate the relationship between these markers and the prognosis of penile cancer in the future.

The management of the regional lymph nodes in penile cancer patients is highly important for long-term survival. However, there is no non-invasive or minimally invasive staging technique that can be used to determine their lymph node status. Proven molecular markers or accurate minimally invasive tests which can be used to identify nodal metastasis are desired.

The present study is associated with some limitations due to its retrospective nature. Our patients received a variety of therapies, including surgery, chemotherapy, radiation therapy, other treatments, and their combinations. Although the treatment options were heterogeneous, we found that the NLR was associated with patient outcomes. Second is that we did not perform mechanistic experiments to determine the roles of neutrophils and/or lymphocytes in penile cancer progression. Nonetheless, the current results support the findings of previous studies indicating correlations between the NLR/inflammation and the clinical outcome of patients with several types of advanced-stage solid tumors. Third, the sample size was low because of the low incidence of penile cancer and the lack of some information, including the degree of extranodal extension [32]. Despite this limitation, the population of the present study represents the largest number of penile cancer patients in whom the NLR was investigated. An additional limitation is that the data in the clinical database study was extracted electronically. Thus, the detailed information about the specific diseases was not confirmed, while the technique allowed us to obtain a large number of cases.

Our immunohistochemistry revealed no significant correlations between the number of tumor-infiltrating CD66b- or CD8-positive immune cells and tumor grade or stage. Nonetheless, higher number of CD66b-positive neutrophils was correlated with lower tumor stage. Wang et al. showed that increased tumor-infiltrating neutrophils and neutrophil-to-lymphocyte ratio in esophageal cancer specimens correlated with disease progression [33]. Although a large number of studies have demonstrated the prognostic value of NLR in various solid tumors, others have failed to show that of tumor-infiltrating neutrophils/lymphocytes in tissue specimens. We indeed performed immunohistochemical staining for CD66b and CD8 in bladder cancer and prostate cancer specimens, but found no significant correlations between the number of immunoreactive immune cells and patient outcomes (unpublished data).

## Conclusion

NLR was found to correlate with lymph node metastasis as well as cancer-specific survival in patients with PSCC. Our data thus suggest that the NLR serves as a biomarker which predicts the patient outcomes.
